# Persisting Verbal Memory Encoding and Recall Deficiency after mGluR5 Autoantibody-Mediated Encephalitis

**DOI:** 10.3390/brainsci13111537

**Published:** 2023-10-31

**Authors:** Niels Hansen, Kristin Rentzsch, Sina Hirschel, Jens Wiltfang, Björn H. Schott, Berend Malchow, Claudia Bartels

**Affiliations:** 1 Department of Psychiatry and Psychotherapy, University Medical Center Göttingen, Von-Siebold-Str. 5, 37075 Goettingen, Germany; sina.hirschel@med.uni-goettingen.de (S.H.); jens.wiltfang@med.uni-goettingen.de (J.W.); bjoernhendrik.schott@med.uni-goettingen.de (B.H.S.); berend.malchow@med.uni-goettingen.de (B.M.); claudia.bartels@med.uni-goettingen.de (C.B.); 2 Clinical Immunological Laboratory Prof. Stöcker, 23627 Groß Grönau, Germany; 3 German Center for Neurodegenerative Diseases (DZNE), Von-Siebold-Str. 3a, 37075 Goettingen, Germany; k.rentzsch@euroimmun.de; 4 Neurosciences and Signaling Group, Institute of Biomedicine (iBiMED), Department of Medical Sciences, University of Aveiro, 3810-193 Aveiro, Portugal; 5 Leibniz-Institute of Neurobiology, University of Magdeburg, 39106 Magdeburg, Germany

**Keywords:** mGluR5 antibodies, autoimmunity, mild cognitive impairment, cognition, encephalitis

## Abstract

Background: Metabotropic glutamate receptors type 5 (mGluR5) play a central role in persistent forms of synaptic plasticity and memory formation. Antibodies to mGluR5 have been reported to be clinically associated with memory impairment. Here, we report on a patient with persistent amnestic cognitive impairment in a single cognitive domain after resolution of mGluR5-associated encephalitis. Methods: We report on the clinical data of a patient in our Department of Psychiatry and Psychotherapy who underwent several diagnostic investigations including a detailed neuropsychological examination, magnetic resonance imaging, and cerebrospinal fluid analysis involving the determination of neural autoantibodies. Results: A 54-year-old woman presented to our memory clinic with pleocytosis 4 months after remission of probable anti-mGluR5-mediated encephalitis, revealing initial pleocytosis and serum proof of anti-mGluR5 autoantibodies (1:32). A neuropsychological examination revealed mild cognitive impairment in verbal memory encoding and recall. The patient received immunotherapy with corticosteroids, and a subsequent cerebrospinal fluid analysis 1.5 months after the onset of encephalitis confirmed no further signs of inflammation. Conclusions: Our results suggest that although immunotherapy resulted in the remission of anti-mGluR5 encephalitis, a verbal memory encoding and recall dysfunction persisted. It remains unclear whether the reason for the persistent verbal memory impairment is attributable to insufficiently long immunotherapy or initially ineffective immunotherapy. Because mGluR5 plays an essential role in persistent synaptic plasticity in the hippocampus, it is tempting to speculate that the mGluR5 antibody–antigen complex could lead to persistent cognitive dysfunction, still present after the acute CNS inflammation stage of encephalitis.

## 1. Introduction

Metabotropic glutamate receptors type 5 (mGluR5), as G protein-coupled receptors, are involved in long-term synaptic plasticity in the hippocampus and play an important role in memory encoding and storage [1,2]. In an animal study [3], mGluR5 was shown to be involved in enhancing synaptic inhibition in the CA1 region of the hippocampus, the central structure for memory formation. Numerous in vivo and in vitro studies demonstrated the role of mGluR5 in electrophysiological and cellular correlates of memory formation such as long-term potentiation and long-term depression in the hippocampus [4]. MGluR5 also plays a role in forming spatial memory [5], as well as in drug memory [6,7] or fear extinction memory [8,9]. Much evidence has emerged in animal experiments on the role of mGluR5 via the use of the strong and selective mGluR5 antagonist 2-methyl-6-(phenylethynyl)-pyridine (MPEP) [4]. Antibodies to mGluR5 may have similar but even more widespread effects as an antagonist of the receptor function. Antibodies to mGluR5 were shown to be involved in memory deficits in humans with encephalitis [10]. MGluR5 encephalitis is a condition clinically characterized by cognitive dysfunction, behavioral abnormalities, mood swings, seizures, and sleep disturbances [11]. In addition, in magnetic resonance imaging (MRI) T2-weighted/fluid-attenuated inversion recovery (FLAIR) sequences, signal abnormalities are frequently and typically observed in mesiotemporal and cortical regions in patients with this clinical presentation [11]. An increase in amygdala volume is also common [11]. Here, we present the first case of a persisting, isolated cognitive deficit after laboratory remission of encephalitis associated with mGluR5 antibodies, suggesting functional impairment of memory-related systems such as the hippocampus. Cognitive deficits have been reported in the mGluR5 encephalitis context, but such a delayed and persistent occurrence is unusual, although no fulminant encephalitis was radiologically evident. To our knowledge, this is the first report of persisting long-term verbal learning and memory deficits in a patient after immunotherapy for anti-mGluR5 encephalitis.

## 2. Case Presentation

**Figure 1:**
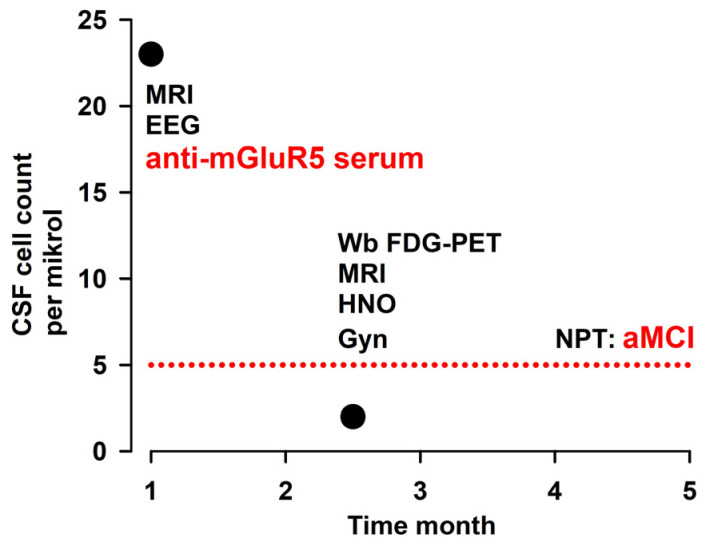
Time course of the number of cerebrospinal fluid cells. Abbreviations: aMCI = amnestic mild cognitive impairment, anti-mGluR5 = anti-metabotropic glutamate receptor 5, CSF = cerebrospinal fluid, EEG = electroencephalography, Gyn = gynecologist, HNO = ear, nose, and throat specialist, MRI = magnetic resonance imaging, NPT = neuropsychological testing, Wb FDG-PET = whole body fluorodesoxyglucose positron emission tomography. Above the dashed red line, CSF cells are pathologically elevated in the CSF.

A 54-year-old female patient presented to our outpatient clinic for autoantibody-mediated psychiatric disorders at the Department of Psychiatry and Psychotherapy in September 2022 (Figure 1). Her only prior diagnosis was arterial hypertension. Apart from her cognitive disorders, her neurological and psychiatric examinations revealed nothing abnormal. Her internal examination revealed no pathologies either. She had probably suffered from autoimmune encephalitis in June 2022. At that time, we detected serum anti-mGluR5 autoantibodies at 1:32 intensity. The presence of MGluR5 autoantibodies was also tested in the CSF, but they were not identified there. An indirect immunofluorescence (IFT) mGluR5 IgG assay was used in Prof. Stöcker laboratory as the test method for mGluR5 autoantibodies in the serum and CSF. The threshold for detecting mGluR5 autoantibody positivity was 1:10. The patient reported having prolonged cephalgia in June 2022, which intensified, together with a fever of about 39 °C (Figure 1).

**Figure 2:**
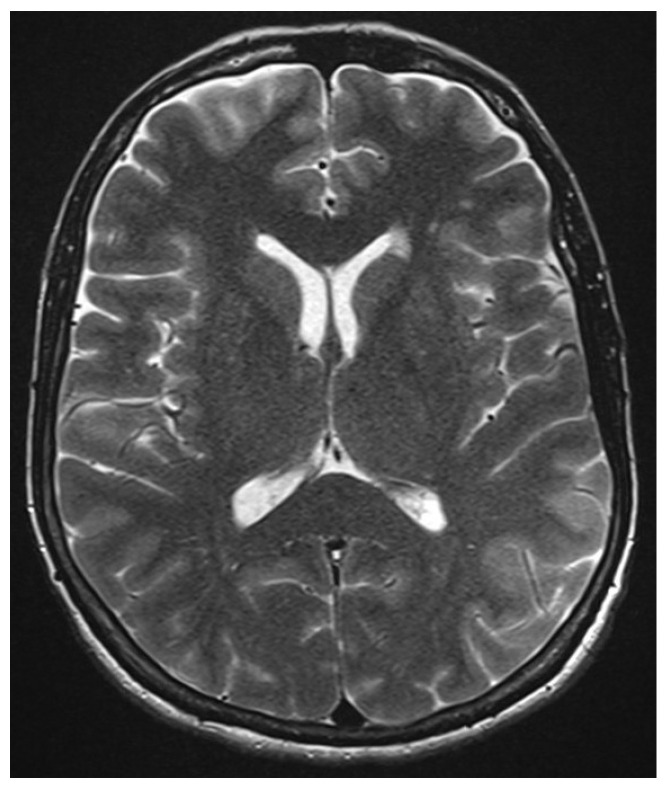
Neuroimaging. Cranial magnetic resonance imaging (cMRI) showed mild cerebral microangiopathy and nonspecific gliosis in the subcortex.

Our female patient was familiar with headaches, but they had been unusually severe, starting in the neck with a stabbing nature, occurring alternately unilaterally or bilaterally or holocephalically. The headaches became significantly weaker 3 months later, responding to ibuprofen. She therefore underwent a differential diagnosis assessment in our neurological clinic in June 2022. Her only other comorbidity was arterial hypertension. Her cranial magnetic resonance imaging (cMRI) in June 2022 (Figure 2) revealed mild cerebral microangiopathy and nonspecific gliosis in the subcortex.

Electroencephalography revealed a left frontotemporal slowing focus. Cerebrospinal fluid (CSF) analysis indicated inflammation evidence and an increased cell count (23 µL, reference, <5µL) (Figure 1). The patient initially underwent a polypragmatic therapy with aciclovir and ceftriaxone for a few days. We were able to terminate this therapy after having detected no pathogens, i.e., measles and rubella viruses, varicella zoster virus (VZV), herpes simplex virus (HSV), Epstein–Barr virus (EBV), cytomegaly virus (CMV). We also conducted tests to determine antibody-specific indexes and multiplex polymerase chain reaction to detect enterovirus ribonucleic acid (RNA), CMV desoxyribonucleic acid (DNA), Escherichia coli DNA, hemophilus influenzae DNA, listeria monocytogenes DNA, Neisseria meningitides DNA, streptococcus agalactiae DNA, HSV1/2 DNA, HPV6 DNA, human parechovirus DNA, VZV DNA, and cryptococcus neoformans DNA. Assuming a diagnosis of autoimmune encephalitis and to relieve her headache, we initiated high-dose methylprednisolone therapy at 500 mg per day for 3 days. The patient’s headache disappeared after this high-dose corticosteroid therapy. We chose to administer a corticosteroid because this class of molecules constitute the first-line therapy for moderate autoimmune encephalitis [12], and the patient presented no contraindications to corticosteroid therapy. According to the Graus criteria [13], the patient suffered from a definite limbic encephalitis, because within 3 months she revealed subacute memory disturbances and new-onset headaches as well as pleocytosis detectable in the CSF and frontotemporal slowing in EEG. There was no evidence of encephalitis on MRI; thus, one criterion was not met. However, with evidence of membrane surface autoantibodies in the serum such as mGluR5 autoantibodies, the second criterion was alternatively fulfilled. We carefully excluded alternative causes. Applying the current Graus criteria [14] for a paraneoplastic neurologic syndrome, we diagnosed a possible paraneoplastic neurologic syndrome with a diagnostic level score of 5, characterized by a high-risk neurological phenotype in the form of limbic encephalitis (score, 3), the presence of autoantibodies, indicating an intermediate-risk phenotype (score, 2), and no evidence of a tumor (non-Hodgkin’s lymphoma). Therefore, a total of 5 points was obtained, suggesting a potential paraneoplastic neurological syndrome.

**Figure 3:**
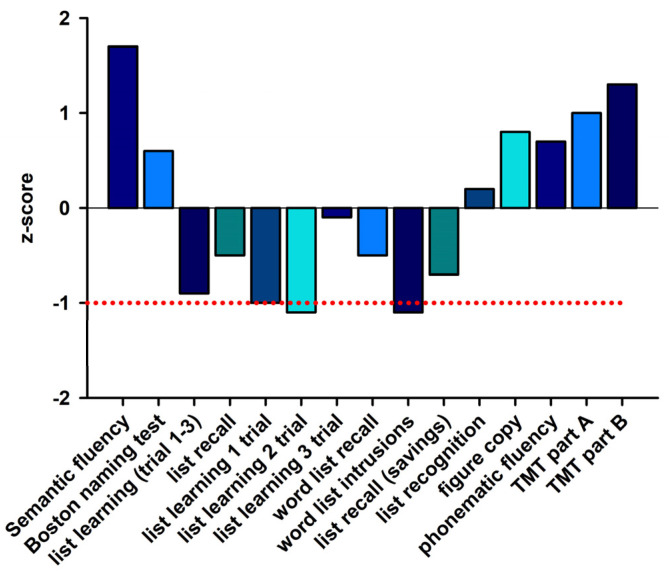
Neuropsychological data. Cognitive tests’ results are presented as z-scores. The red dashed line indicates the unique standard deviation. TMT = trial making test.

The patient’s initial response in terms of headache and signs of inflammation in the CNS (regressive pleocytosis) suggested a good response to corticosteroids. We therefore saw no need to escalate to plasmapheresis therapy. Nor was there any need for IVIG, considering the patient’s response to corticosteroids. The patient underwent lumbar puncture 1.5 months later, and her cell count then normalized (2 µL). Autoantibodies were retested in the serum, but not in the CSF, 3.5 month later, but were not detected. This did not exclude the possibility that mGluR5 autoantibodies had bound to brain structures after a presumed opening of the blood–brain barrier during encephalitis and were therefore no longer detectable in the serum. A whole-body positron emission computed tomographic scan was performed to search for tumors, which revealed unremarkable brain findings. Thus, the lack of abnormalities on PET imaging in the brain such as an anterior–posterior glucose metabolism gradient typical of anti-NMDAR encephalitis [15] and temporomesial hypermetabolism typical of LGI1 antibody-mediated encephalitis [16] did not additionally support a limbic encephalitis diagnosis, though it did not argue against it either. The patient’s tonsils showed a probable mild reactive inflammatory anomaly. Her gynecologic sonography (after an abnormal finding in the left breast) was also unremarkable. She reported having suffered mild recurrent herpes infections (on the eye and lips) and sometimes feeling a slight lack of energy and lability of affect. In June 2022, she was in a depressed mood, which completely receded. She returned to work for a nursing service but noticed memory problems. Given the headaches and affective lability as well as depressive symptoms, together with CSF signs of inflammation and the serum detection of mGluR5 antibodies, we concluded that her headache was likely triggered by anti-mGluR5-associated encephalitis in June 2022. A neuropsychological examination in September following a mild memory impairment revealed evidence of an amnestic mild cognitive impairment (aMCI) in a single domain. The single domain consisted of an encoding impairment and a delayed recall of complex verbal content (Figure 3).

The other cognitive parameters we tested were consistent with the patient’s educational level, and included orientation, semantic word fluency, phonematic word fluency, confrontation naming, visuomotor coordination, cognitive processing speed, switching ability, divergent thinking and reactive cognitive fluency, action planning, working and figural memory, encoding and consolidation of verbal non-associated information, visuoconstructive skills, and visual spatial perception. Her profile was therefore consistent with an aMCI in a single cognitive domain (Figure 3). Two months later, at a follow-up visit, the patient continued to complain of mild cognitive deficits that were less severe but persisting. Our differential diagnosis of her persistent MCI indicated the preliminary stage of a neurodegenerative disease. We could not entirely rule out an MCI caused by AD, but this was unlikely, given the absence of AD dementia biomarkers (no reduced Aβ42/40 ratio and no elevated ptau181 level). Another neurodegenerative disease was also unlikely because of the absence of additional clinical features. Other causes of cognitive impairment such as vitamin deficiencies or infectious diseases were also excluded. Likewise, as the patient’s depressive symptoms subsided, we could not rely on them as a possible factor for persistent MCI. Her prognosis is quite encouraging, as she is already reporting improvement; thus, we do not suspect a conversion to dementia.

## 3. Discussion

To the best of our knowledge, the novelty of our report is the finding that dysfunctional verbal memory encoding and recall can persist, and aMCI may develop after autoimmune encephalitis with mGluR5 autoantibodies has resolved. There is case-based evidence of cognitive impairment as a frequent clinical feature along with behavioral abnormalities in patients with mGluR5 autoantibody-mediated encephalitis [11,17,18]. However, to date, anti-mGluR5 encephalitis accompanied by headache as the main symptom and regressing acute inflammatory encephalitis with completely normalized pleocytosis have not been described as triggering a delayed-onset verbal memory impairment without further cognitive abnormalities. Such late-onset verbal memory impairment may be related to an expired or persistent immune response in the hippocampus, since mGluR5 is present in the hippocampus and cortex [19], and the hippocampal subfields and hippocampal networks play a particularly important role in forming verbal memory [20,21].

Animal studies indicated that inhibitory gamma-amino butyric acid (GABAergic) synapses containing mGluR5 are regulated by endogenous glutamate [3]. Autoantibodies against mGluR5 could thus affect GABAergic inhibition and cause cognitive impairment. Nevertheless, it is unclear how cognitive impairment can be explained in the absence of mGluR5 antibodies after resolved autoimmune encephalitis. The absence of mGluR5 antibodies in the serum may imply that mGluR5 autoantibodies still cause functional deficits in the brain because the brain acts as an immunoprecipitator, and the mGluR5 antibodies deposited in the hippocampus cause impaired hippocampal GABAergic synaptic transmission. We know that mGuR5 is essential for persistent forms of synaptic plasticity in rodents [2], indicating that a transient or persistent blockage of these receptors may cause hippocampus-based encoding and memory storage impairments. Although no mGluR5 antibodies were found in the patient’s CSF fluid, these autoantibodies might have entered her brain through a temporarily disrupted blood–brain barrier and then blocked mGluR5 where it is highly expressed, such as in the hippocampus [22]. Such a brief opening of the blood–brain barrier might have caused mGluR5 autoantibodies in the CSF to disappear, because the brain can function as an immunoprecipitator. This was demonstrated in conjunction with the presence of NMDAR autoantibodies in a study [23]. mGluR5 also plays an important role in encoding information in the hippocampal CA3 region by shifting the threshold for long-term potentiation in associational–commissural CA3 synapses, reflecting a shift that alters the signal-to-noise ratio during information encoding [1]. The CA3 region of the hippocampus is extremely important for memory encoding, processing input information from the prior relay station dentate gyrus [24]; so, the blockage of mGluR5 by autoantibodies could disrupt this information processing, thus explaining the occurrence of persisting learning deficits and difficulties in remembering verbal information. In particular, the patient’s impairment in the ability to encode and retrieve verbal information after anti-mGluR5 encephalitis might have resulted from hippocampal disruption of engram formation by persistent mGluR5 autoantibodies or a permanent neuronal functional damage in the hippocampus after mGluR5 blockade by mGluR5 autoantibodies. Studies investigating long-term cognitive functioning found that 52% of the patients with autoimmune encephalitis still suffered cognitive impairment 20 months after their encephalitis onset [25]. An over-60-day delay in immunotherapeutic or tumor treatment proved to be more likely associated with cognitive impairment at the last follow-up [25]. Early therapy is therefore essential to prevent long-term cognitive impairment in patients suffering from autoimmune encephalitis. Nevertheless, early immunotherapy obviously failed to prevent our patient’s aMCI from developing later. In another study [26] of 33 patients, the majority (94%) had improved at their last follow-up, but 6% of the patients continued to suffer from residual symptoms such as mood swings and cognitive dysfunction. Advanced age is an acknowledged factor correlating with poor outcome [26] but did not concern our patient. Therefore, a discussion is warranted on whether further immunotherapy would have alleviated her persistent cognitive impairment. After a benefit–risk assessment at that timepoint, we opted not to pursue further immunotherapy with corticosteroids or rituximab, especially since the inflammatory activity in her CNS had regressed, and further immunotherapy would have been of negligible benefit. Nonetheless, in the presence of persistent MCI, consideration is warranted as to whether chronic immunotherapy is beneficial, assuming a persistent mGluR5-dependent immune response in the brain.

## 4. Limitations

As a weakness of our report, note that the evidence level of our results is low and that we cannot guarantee that our autoantibody findings were not related to our patient’s subsequent cognitive impairment; we agree that this finding may be incidental. However, anti-mGluR5 encephalitis is often associated with cognitive impairment clinically. Note that MCI occurred within three months after we detected mGluR5 autoantibodies, and autoantibodies against membrane surface antigens such as mGluR5 are frequently pathogenic. Therefore, the clinical correlate of persisting MCI may be attributable to an immune reaction involving mGluR5 autoantibodies, although as a further limitation, we must add that we have no evidence for this assumption. However, the moderate CNS inflammation associated with cognitive symptoms after the patients suffered from the encephalitic disease argue against such a claim. We believe it is unlikely that immunotherapy itself led to impaired cognition as a side effect. What is more probable is that immunotherapy was not administered early enough at an adequate intensity or long enough, and this may have caused the patient’s permanent functional impairment in verbal memory formation. Another limitation is that mGluR5 autoantibodies were not present in the CSF and were not tested 1.5 months later in the CSF, at the time of remission of pleocytosis. There is therefore little evidence for the possible affection of immune processes in the brain, but it is quite possible that the mGluR5 autoantibodies had already bound to brain structures such as the hippocampus and were therefore not detectable in the CSF.

## 5. Conclusions

Overall, we described a potentially pathophysiological mechanism of how mGluR5 antibody-mediated encephalitis may have caused our patient’s persistent aMCI. The strength of our report is the self-reported cognitive impairment that developed after initial remission of mGluR5 autoantibody-associated encephalitis. Another limitation of our report, besides those reported above, is that it regarded a single case. It is therefore imperative that more research be conducted in patients with GluR5 autoantibody-mediated encephalitis to monitor cognitive impairment over the long term.

## Data Availability

Data are available from the corresponding author on demand.
