# Correction: Hansen et al. Persisting Verbal Memory Encoding and Recall Deficiency after mGluR5 Autoantibody-Mediated Encephalitis. *Brain Sci.* 2023, *13*, 1537

**DOI:** 10.3390/brainsci14030287

**Published:** 2024-03-18

**Authors:** Niels Hansen, Kristin Rentzsch, Sina Hirschel, Jens Wiltfang, Björn H. Schott, Berend Malchow, Claudia Bartels

**Affiliations:** 1 Department of Psychiatry and Psychotherapy, University Medical Center Göttingen, Von-Siebold-Str. 5, 37075 Göttingen, Germany; sina.hirschel@med.uni-goettingen.de (S.H.); jens.wiltfang@med.uni-goettingen.de (J.W.); bjoernhendrik.schott@med.uni-goettingen.de (B.H.S.); berend.malchow@med.uni-goettingen.de (B.M.); claudia.bartels@med.uni-goettingen.de (C.B.); 2 Clinical Immunological Laboratory Prof. Stöcker, 23627 Groß Grönau, Germany; k.rentzsch@euroimmun.de; 3 German Center for Neurodegenerative Diseases (DZNE), Von-Siebold-Str. 3a, 37075 Göttingen, Germany; 4 Neurosciences and Signaling Group, Institute of Biomedicine (iBiMED), Department of Medical Sciences, University of Aveiro, 3810-193 Aveiro, Portugal; 5 Leibniz-Institute of Neurobiology, University of Magdeburg, 39106 Magdeburg, Germany

**Affiliations:** In the published publication [1], there was an error regarding the affiliations for Niels Hansen, Kristin Rentzsch, and Jens Wiltfang. The updated affiliations should include Niels Hansen, Department of Psychiatry and Psychotherapy, University Medical Center Göttingen, Von-Siebold-Str. 5, 37075 Göttingen, Germany; Kristin Rentzsch, Clinical Immunological Laboratory Prof. Stöcker, 23627 Groß Grönau, Germany; and Jens Wiltfang, Department of Psychiatry and Psychotherapy, University Medical Center Göttingen, Von-Siebold-Str. 5, 37075 Göttingen, Germany; the German Center for Neurodegenerative Diseases (DZNE), Von-Siebold-Str. 3a, 37075 Göttingen, Germany; and the Neurosciences and Signaling Group, Institute of Biomedicine (iBiMED), Department of Medical Sciences, University of Aveiro, 3810-193 Aveiro, Portugal. The authors state that the scientific conclusions are unaffected. This correction was approved by the Academic Editor. The original publication has also been updated.
